# Supplementary material for: Somatic point mutations are enriched in non-coding RNAs with possible regulatory function in breast cancer
Source: Commun Biol. 2022 Jun 7;5:556. doi: 10.1038/s42003-022-03528-0 (PMC9174258; doi:10.1038/s42003-022-03528-0)
Supplement: Supplementary file 2 — Supplementary Information [file 42003_2022_3528_MOESM2_ESM.pdf]

Somatic point mutations are enriched in non-coding RNAs with possible regulatory function in breast cancer

Narges Rezaie[1#], Masroor Bayati[2#], Mehrab Hamidi[2], Maedeh Sadat Tahaei[2], Sadegh Khorasani[2], Nigel H. Lovell[3], James Breen[4,5,6], Hamid R. Rabiee*[2], Hamid Alinejad-Rokny* [7,8,9]

[1] Center for Complex Biological Systems, University of California Irvine, California, 92697, US

[2] Bioinformatics and Computational Biology Lab, Department of Computer Engineering, Sharif University of Technology, Tehran, 11365, IR

[3] Tyree Institute of Health Engineering and The Graduate School of Biomedical Engineering, UNSW Sydney, Sydney, NSW, 2052, AU

[4]South Australian Health & Medical Research Institute, Adelaide SA 5000, AU

[5] Robinson Research Institute, University of Adelaide, SA 5006, AU

[6] Bioinformatics Hub, University of Adelaide, Adelaide SA 5006, AU

[7] BioMedical Machine Learning Lab (BML), The Graduate School of Biomedical Engineering, UNSW Sydney, Sydney, NSW, 2052, AU

[8] UNSW Data Science Hub, The University of New South Wales (UNSW Sydney), Sydney, NSW, 2052, AU

[9] Health Data Analytics Program, AI-enabled Processes (AIP) Research Centre, Macquarie University, Sydney, 2109, AU

These authors contributed equally.

* To whom correspondence should be addressed. Tel: +61 2 9385 3911; e-mails: h.alinejad@unsw.edu.au , rabiee@sharif.edu

SUPPLEMENTARY FIGURES

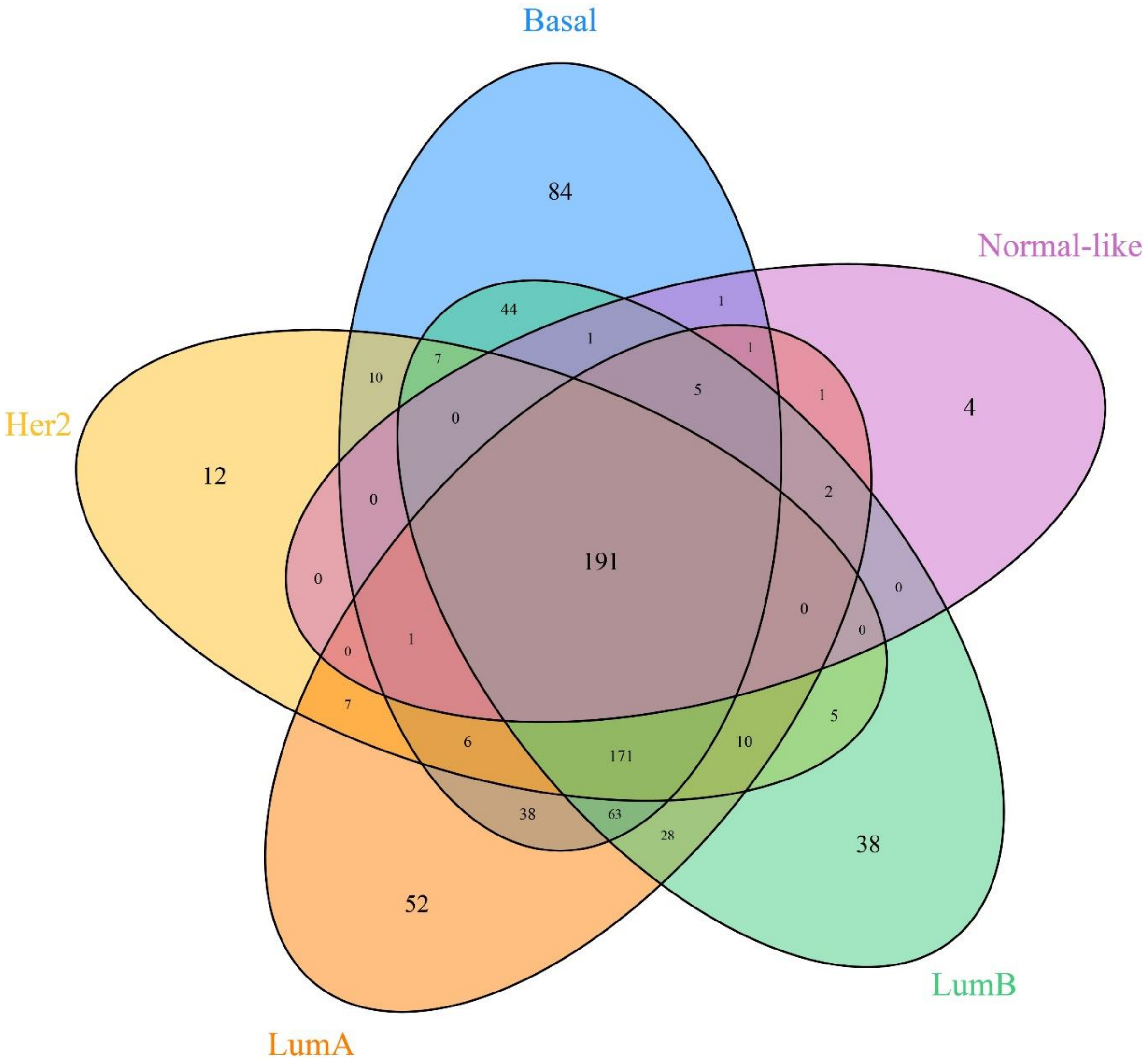

Figure S1. Number of significant ncRNAs that were mutated in breast cancer subtypes. We obtained PAM50 subtype annotation of 346 ICGC breast cancer samples from a publication by Nik-Zainal *et al*. [30]. In our list of significant ncRNAs, 782 hits had mutations in the above-mentioned samples. Most ncRNAs were mutated in samples of multiple subtypes, however, we also observed that each subtype had a unique set of mutated ncRNAs that were not mutated in other subtypes. Find the list of subtype-specific ncRNAs in **Supplementary Data 2**.

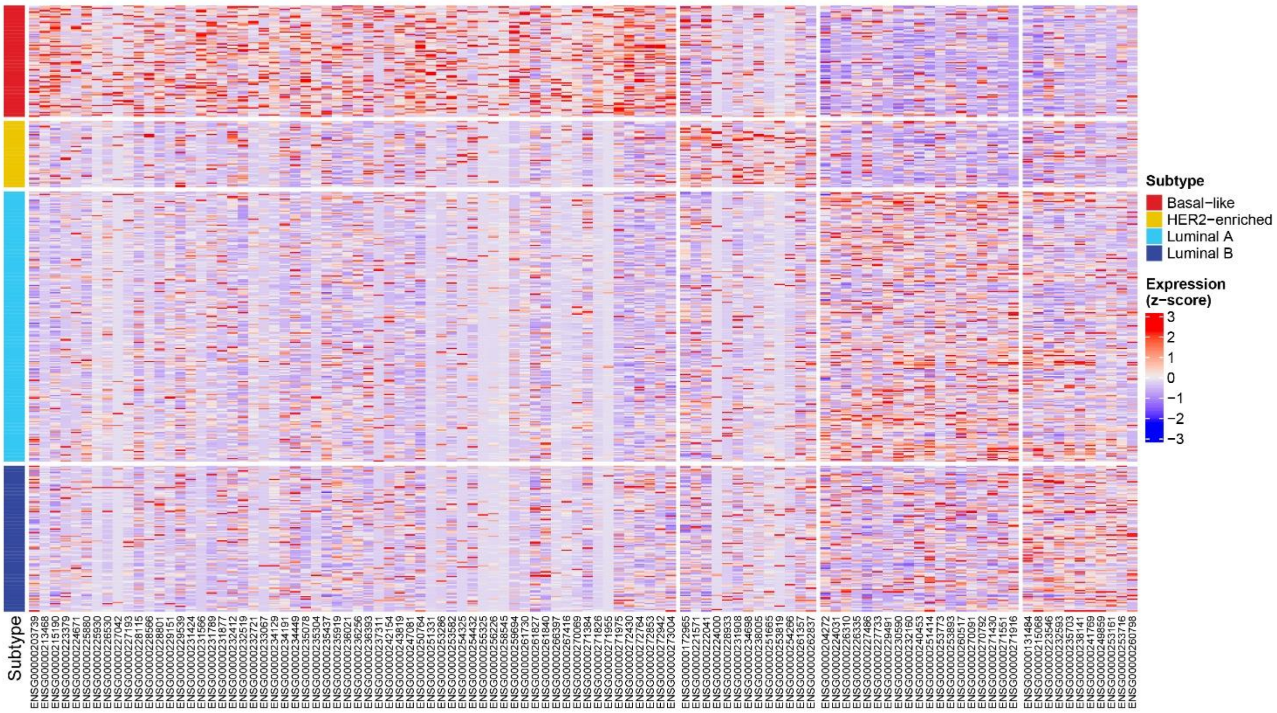

Figure S2. Differentially expressed ncRNAs across TCGA-BRCA tumors (expression profiles from TANRIC). 504 ncRNAs of the 1030 significant ncRNAs were found in the TANRIC gene expression list. Although the 504 ncRNAs were expressed ubiquitously across all breast cancer patients, we had however 106 ncRNAs that were differentially expressed between the breast cancer subtypes. The 106 ncRNAs show some specificity in breast cancer subtypes. A list of these differentially expressed genes is provided in **Supplementary Data 3**.

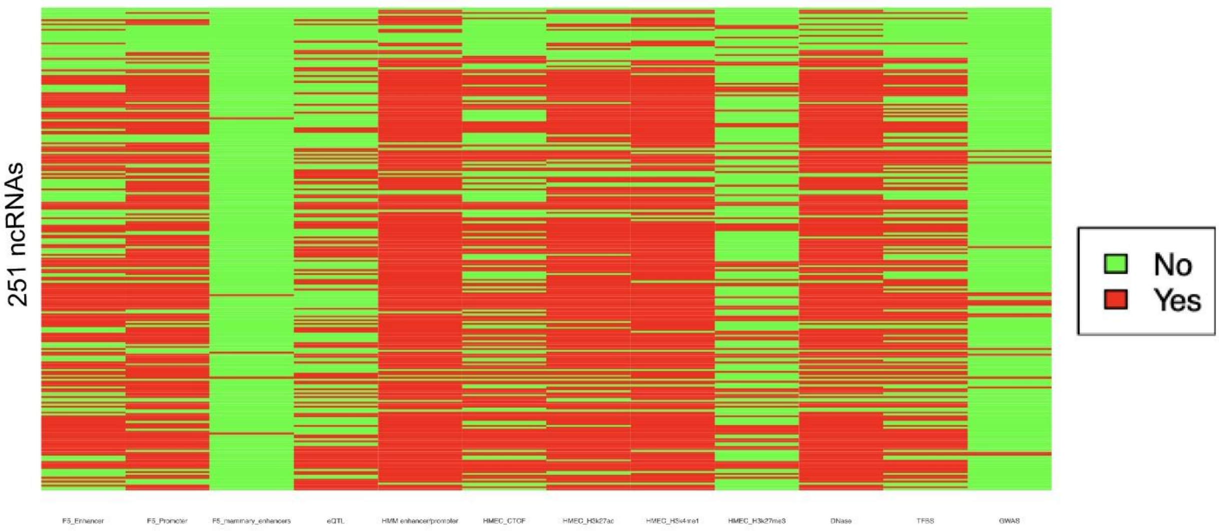

Figure S3. Overlapping of regulatory features with 251 BC-associated ncRNAs that have multiple Hi-C based assays support. This figure shows the overlapping of 251 candidate ncRNAs with genomic and epigenetic features, GWAS and eQTL SNPs used in this study.

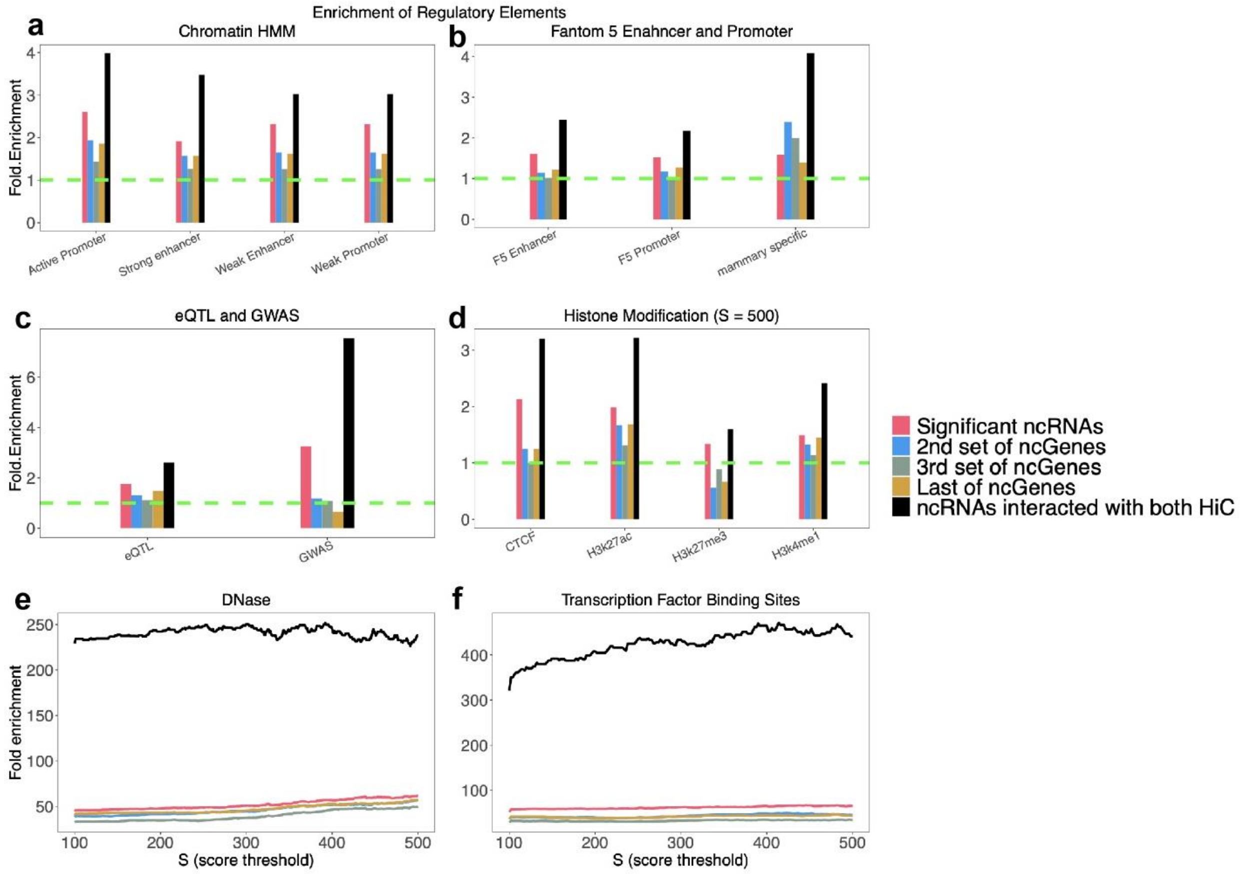

Figure S4. Enrichment of regulatory features in 251 BC-associated ncRNAs with multiple Hi-C based assays support. As figure shows the 251 BC-associated ncRNAs have much higher enrichment of overlapping with regulatory features, GWAS and eQTL polymorphisms.

	Having mutation in this ncRNA	
Being breast cancer sample	# breast cancer samples which have mutation in this ncRNA	# breast cancer samples which do not have mutation in this ncRNA
	# non-breast cancer samples which have mutation in this ncRNA	# non-breast cancer samples which do not have mutation in this ncRNA

Figure S5. The contingency table used for Fisher's exact test. For each ncRNA, a contingency table is constructed based on this scheme to perform a Fisher's exact test to determine if the corresponding non-coding RNA is significantly mutated in breast cancer samples compared to other cancer types.

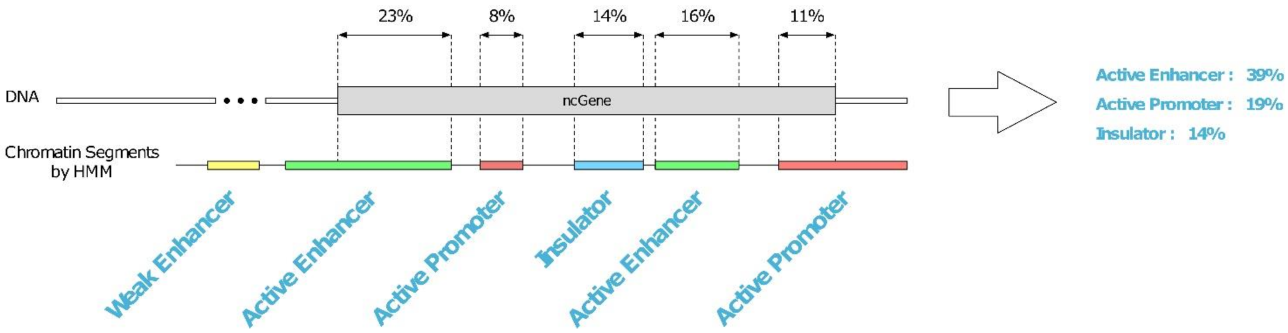

Figure S6. Overlapping between a non-coding RNA and chromatin segments. This diagram shows an example situation in which a non-coding RNA overlaps with different types of segments in chromHMM annotation. The percentage of overlap with each segment is calculated as the total number of nucleotides in the non-coding RNA region covered by each type of segment divided by the non-coding RNA length.
