# Supplementary material for: Somatic point mutations are enriched in non-coding RNAs with possible regulatory function in breast cancer
Source: Commun Biol. 2022 Jun 7;5:556. doi: 10.1038/s42003-022-03528-0 (PMC9174258; doi:10.1038/s42003-022-03528-0)
Supplement: Supplementary file 3 — Description of Additional Supplementary Files [file 42003_2022_3528_MOESM3_ESM.pdf]

Description of Additional Supplementary Files

File name: Supplementary Data 1

Description**: Significantly mutated ncRNAs.** A list of non-coding RNAs that are significantly mutated in breast cancer samples compared to samples with other cancers (P-value < 0.05).

File name: Supplementary Data 2

Description**: Mutational status of significantly mutated ncRNAs in breast cancer subtypes across 346 ICGC breast cancer samples.** We obtained PAM50 subtype annotation of 346 ICGC breast cancer samples from a publication by Nik-Zainal *et al*.

File name: Supplementary Data 3

Description**: Significantly mutated ncRNAs that are differentially expressed in breast cancer subtypes across 501 TCGA breast cancer samples.** We downloaded the expression dataset from Breast invasive carcinoma (BRCA) gene expression matrix from TANRIC.

File name: Supplementary Data 4

Description**: Annotating candidate ncRNAs with BC-associated GWAS SNPs and breast tissue-related eQTL polymorphisms.** Annotating significantly mutated ncRNAs with breast cancer-related GWAS SNPs. GWAS SNPs were downloaded from EBI GWAS Catalog and GWASdb v2 from Wang Lab. All GWAS SNPs with a P-value less than 1e-8 were considered. We also used GTEx breast-related eQTL polymorphisms to identify how many of our candidate ncRNAs encompass at least one breast tissue-related eQTL polymorphism.

File name: Supplementary Data 5

Description**: Annotating candidate ncRNAs with ENCODE chromHMM predicted chromatin states.** Annotating significantly mutated ncRNAs (candidate ncRNAs) with ENCODE predicted chromatin state marks (chromHMM) presented in the HMEC cell line.

File name: Supplementary Data 6

Description**: Annotating candidate ncRNAs with FANTOM5 features.** Annotating significantly mutated ncRNAs (candidate ncRNAs) with FANTOM5 promoters, enhancers, and FANTOM5 breast differentially expressed enhancers.

File name: Supplementary Data 7

Description**: List of candidate ncRNAs that overlapped with both ENCODE and FANTOM5 enhancer/promoter signals.** 317 candidate ncRNAs overlapped with both ENCODE and FANTOM5 enhancer marks. 257 candidate ncRNAs overlapped with both ENCODE and FANTOM5 promoter marks.

File name: Supplementary Data 8

Description**: Annotating candidate ncRNAs with histone marks.** Annotating significantly mutated ncRNAs (candidate ncRNAs) with HMEC related histone modification, including CTCF, H3K27ac, H3K4me1, and H3K4me3. For each category and each gene, we identified the percentage of overlap and average score as described in the method section.

File name: Supplementary Data 9

Description**: Annotating candidate ncRNAs with DHS and transcription factors.** The list of significantly mutated ncRNAs (candidate ncRNAs) with HMEC-related DHSs and transcription factors. For each gene and each category, we calculated the percentage and score of the overlapping. The name of each category is shown in the "Details for DNase Clusters" sheet.

File name: Supplementary Data 10

Description**: Annotating ncRNAs with HMEC related Hi-C interactions.** We used chromosome conformation capture data (Hi-C) as the evidence of potential enhancer activity for genomic regions encompassing candidate ncRNAs. We used two replications of HMEC-related Hi-C data from the Rao *et al*. study. We only reported significantly interacting regions for each replication. We identified ncRNA-coding gene pairs, in which the ncRNA overlapped with one side of the interaction and coding gene overlapped with another side. We then annotated the ncRNA side of the interactions with breast-related regulatory features.

File name: Supplementary Data 11

Description**: Same as Supplementary Data 10, but only common interactions are considered.** We only considered those Hi-C interactions that appeared as significant interactions in both replications (common interactions between SRR1658680 and SRR1658686-9). Common interactions also filtered out if neither left nor right sides of the interaction overlapped with our candidate ncRNAs.

File name: Supplementary Data 12

Description**: Same as Supplementary Data 11, but we considered those interactions that become significant in either Hi-C replicate1 or replicate 2.** We considered those Hi-C interactions that appeared as significant interactions in at least one of the replications. Significant interactions also filtered out if neither left nor right sides of the interaction overlapped with our candidate ncRNAs.
